# Supplementary material for: A fine-scale Arabidopsis chromatin landscape reveals chromatin conformation-associated transcriptional dynamics
Source: Nat Commun. 2024 Apr 16;15:3253. doi: 10.1038/s41467-024-47678-7 (PMC11021422; doi:10.1038/s41467-024-47678-7)
Supplement: Supplementary file 1 — Supplementary Information [file 41467_2024_47678_MOESM1_ESM.pdf]

Supplementary Information for

A fine-scale *Arabidopsis* chromatin landscape reveals chromatin conformation-associated transcriptional dynamics

Yueying Zhang[1,2†], Qianli Dong[1†], Zhen Wang[2,3†], Qinzhe Liu[4,5†], Haopeng Yu[2], Wenqing Sun[1], Jitender Cheema[2], Qiancheng You[4,5], Ling Ding[1], Xiaofeng Cao[3], Chuan He[4,5], Yiliang Ding[2*], Huakun Zhang[1*]

1. Key Laboratory of Molecular Epigenetics of Ministry of Education, Northeast Normal University, Changchun 130024, China

2. Department of Cell and Developmental Biology, John Innes Centre, Norwich Research Park, Norwich NR4 7UH, United Kingdom

3. Institute of Genetics and Developmental Biology, Chinese Academy of Sciences, Beijing, 100101, China

4. Howard Hughes Medical Institute, The University of Chicago, Chicago, IL 60637, USA.

5. Department of Chemistry, Department of Biochemistry and Molecular Biology, Institute for Biophysical Dynamics, The University of Chicago, Chicago, IL 60637, USA.

* Correspondence to: Yiliang Ding (yiliang.ding@jic.ac.uk), and Huakun Zhang (zhanghk045@nenu.edu.cn)

† These authors contributed equally to this work.

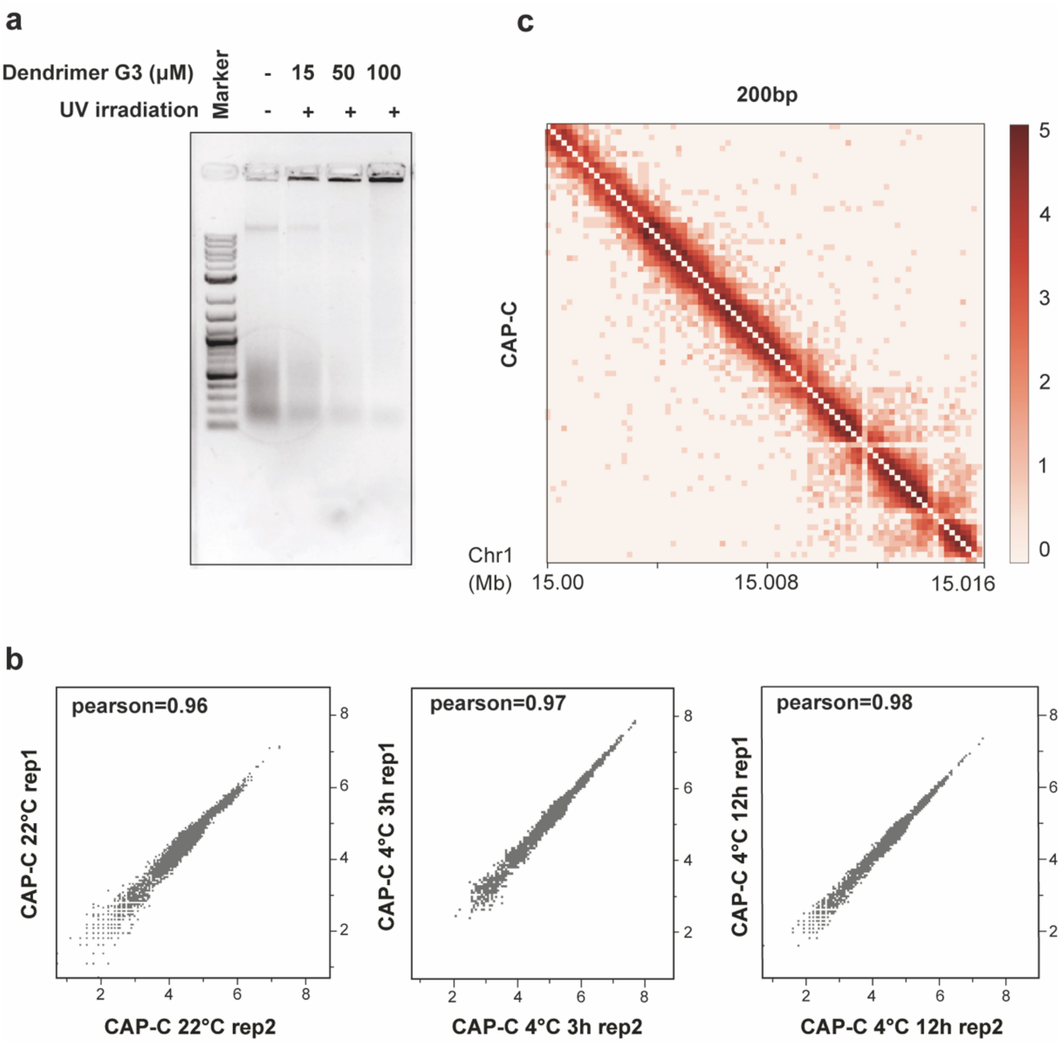

Supplementary Fig. 1 | The validation and reproducibility of *Arabidopsis* CAP-C libraries.

(a) Psoralen-functionalized dendrimer G3 was subjected to serial dilution in concentrations of 100, 50, and 15 µM. The diluted solution was then diffused into fixed cell nuclei, which were exposed to UV irradiation for 30 minutes. The resulting DNA-dendrimer complexes were identified by a shift in the band towards the top.

(b) The reproducibility of G3 contact maps from *Arabidopsis* seedlings at 22°C and after 3 hours or 12 hours of cold treatments (4°C) is shown using scatter plots. Pairwise scatter plots comparing contacts between each replicate were plotted using hicCorrelate from HiCExplorer[1]. The hicCorrelate of CAP-C contact maps suggests a high level of reproducibility between each biological replicate.

(c) CAP-C contact matrices at 200 bp resolution for Chr1, 15.00Mb-15.016 Mb corresponding to the region in Fig.1c.

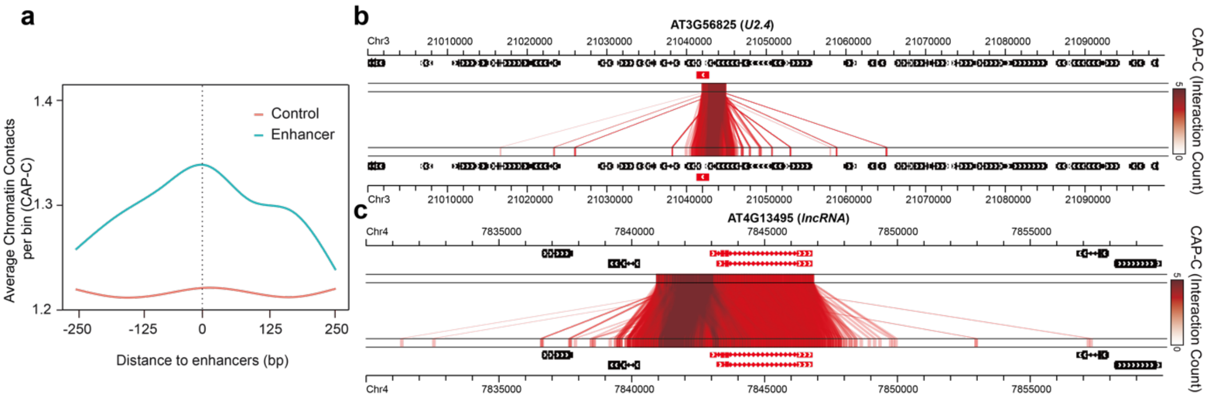

Supplementary Fig. 2 | Validation of CAP-C chromatin contacts at the gene level.

(a) Chromatin contacts enriched at active enhancer loci. CAP-C chromatin contacts across 250 bp upstream and downstream of the enhancer region (green line) show a discernible peak. There is no peak in the randomized data set (red line).

(b and c) GIVE plots showing CAP-C contacts involving *AT3G56825* (U2.4) (b) and *AT4G13495* (*lncRNA*) (c). For displaying contacts, the chromosomes are plotted horizontally twice, to give an upper track and a lower track. Each line in the lower track represents a chromatin contact; the colour scale is proportional to contact strength: the darker the colour, the stronger the contact. The black boxes and lines (upper track) indicate the position of genes on the chromosomes.

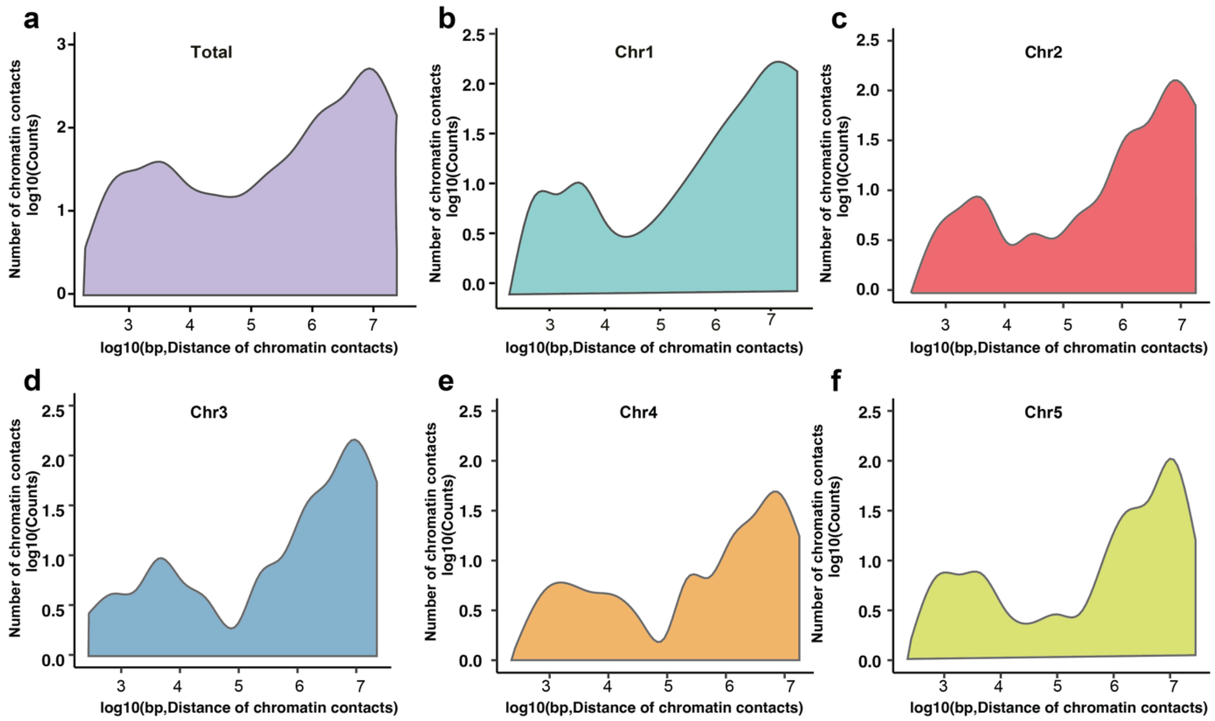

Supplementary Fig. 3 | The distribution of the distances for E-P chromatin contacts in all chromosomes.

(a-f) The distribution of the distances for all E-P chromatin contacts in all five chromosomes are plotted respectively: Total E-P chromatin contacts for all chromosomes (**a**) and E-P chromatin contacts in each individual chromosome (**b-f**).

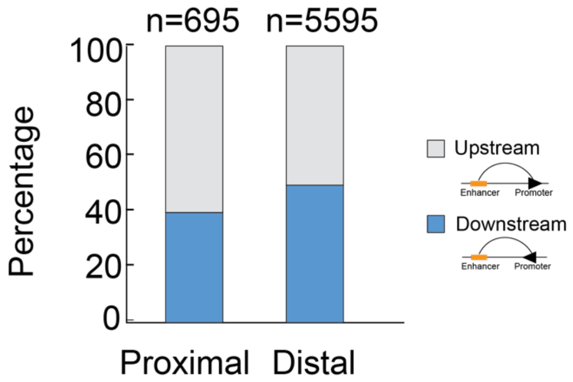

Supplementary Fig. 4 | Proportions of different types of E-P chromatin contacts.

The proportion of enhancers upstream and downstream of the connected promoters in both proximal and distal E-P chromatin contacts.

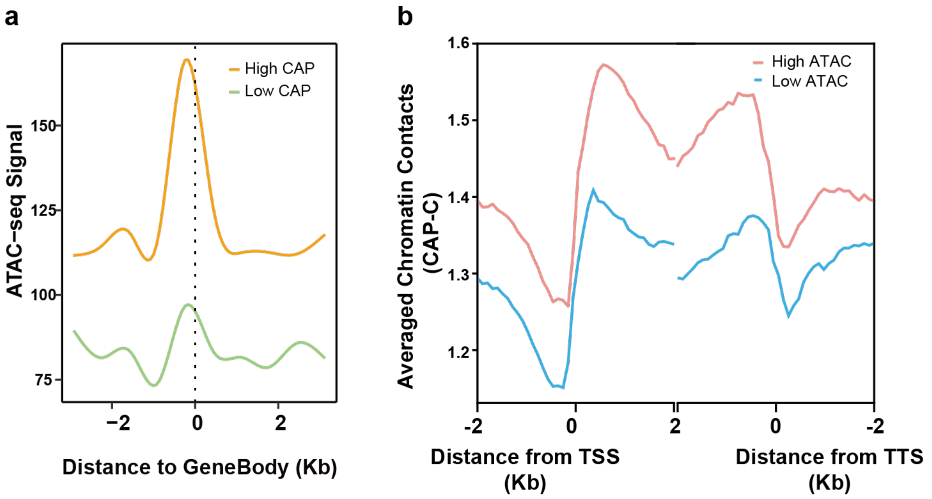

Supplementary Fig. 5 | CAP-C association between chromatin accessibility signals and chromatin contacts at gene locus level.

(**a**) Genes were categorized based on the enrichment of CAP-C data across the gene body. The 10% of genes with the highest level of CAP-C enrichment (represented by orange line) and the lowest level of CAP-C enrichment (represented by green line) were used to plot ATAC-seq signal profiles[2] within the 3Kb flanking region of the gene body, n=2648.

(**b**) Meta-profile shows CAP-C interaction signal profiles with high (red) and weak (blue) ATAC-seq signal enrichment, n=2682. Genes were aligned by the transcription start site (TSS) and the transcription termination site (TTS).

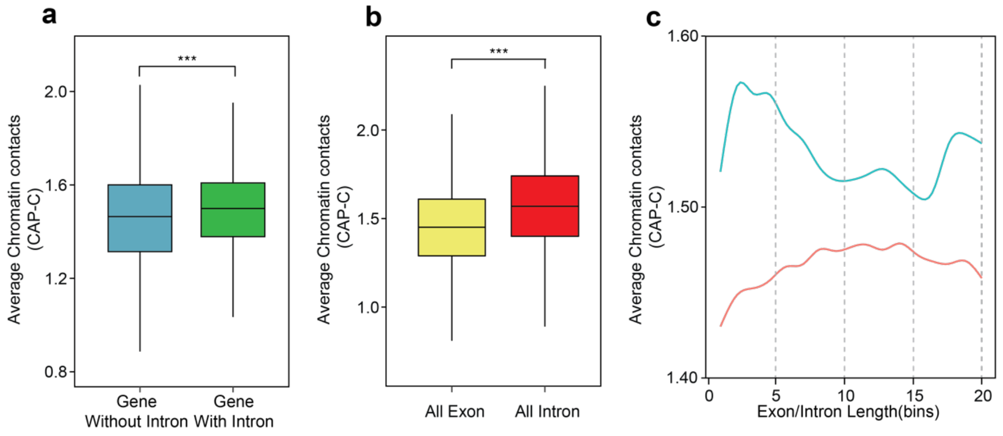

Supplementary Fig. 6 | Chromatin contact characteristics across genes.

(a) Box plot showing the chromatin contacts for genes with (n = 22111) and without introns (n = 5334). *p* values: ***, $p < 0.001$. Two-sided Wilcoxon rank sum exact test.

(b) Box plot showing the chromatin contacts across all exons and introns of all genes. For exons, n = 146346, for introns, n = 118901, *p* values: ***, $p < 0.001$. Two-sided Wilcoxon rank sum exact test.

(c) The distribution of chromatin contacts inside introns (blue) and exons (red). Each exon or intron was divided into 20 bins. Each bin shows the average number of chromatin contacts across each corresponding bin.

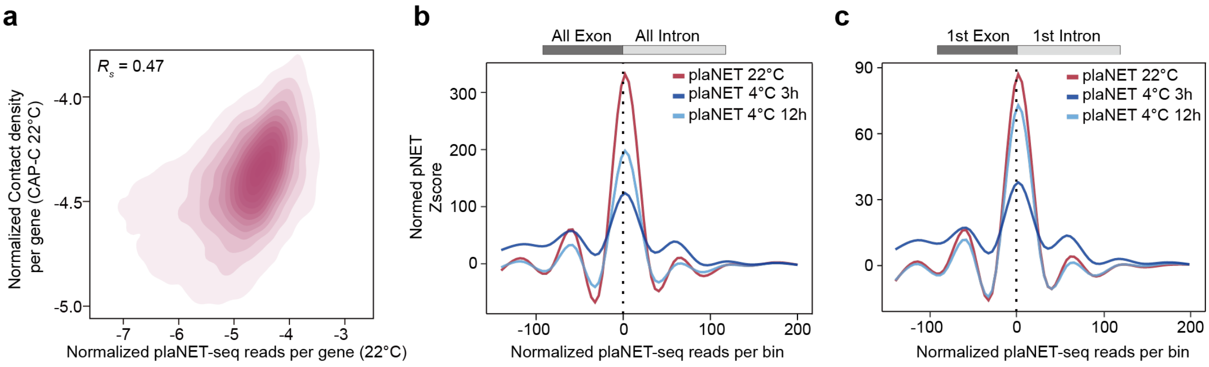

Supplementary Fig. 7 | Correlations between chromatin contacts and Pol II densities, and Pol II densities across 5'SS under three temperature treatments

(a) Correlation between gene chromatin contacts and transcriptional activities at 22°C. The gene chromatin contact was measured by normalized chromatin contact density per gene. The transcription activity was measured by the plaNET-seq[3]. The correlation between gene chromatin contacts and Pol II activities was 0.47, n = 22910, p value < 1.7e-308, based on two-sided Spearman's rank correlation. **(b and c)** The line plot shows the plaNET-seq Pol II density profile 100 bp upstream and 200 bp downstream of the (**b**) 5'SS or (**c**) the 1st 5'SS (22°C in red, 4°C treatment for 3h in dark blue, 4°C treatment for 12h in light blue). The 10-bp smoothing was performed in generating the continuous lines.

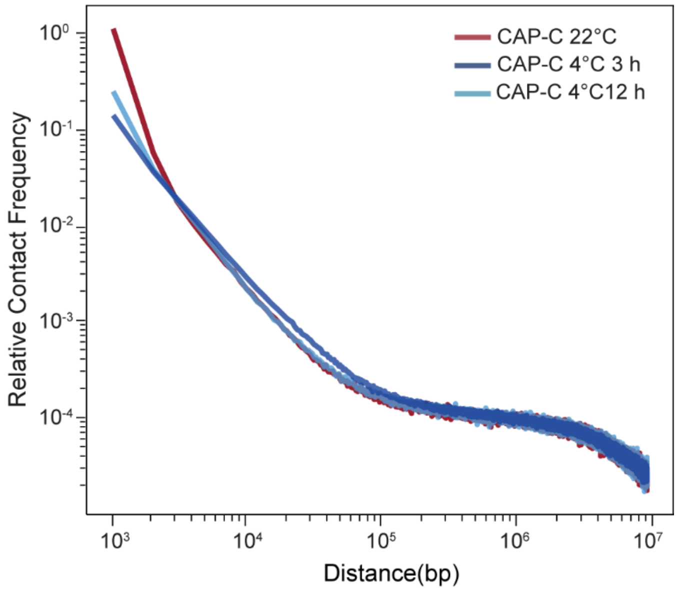

Supplementary Fig. 8 | Relative contact frequency versus genomic distance curves of CAP-C under different temperature conditions.

X-axis: the distance between contact loci from 1 kb to 10 Mb. Y-axis: contact density normalized by sequencing depth. The red line, dark blue line and light blue line represent CAP-C performed under 22°C, 4°C treatment for 3 hours, and 4°C treatment for 12 hours, respectively.

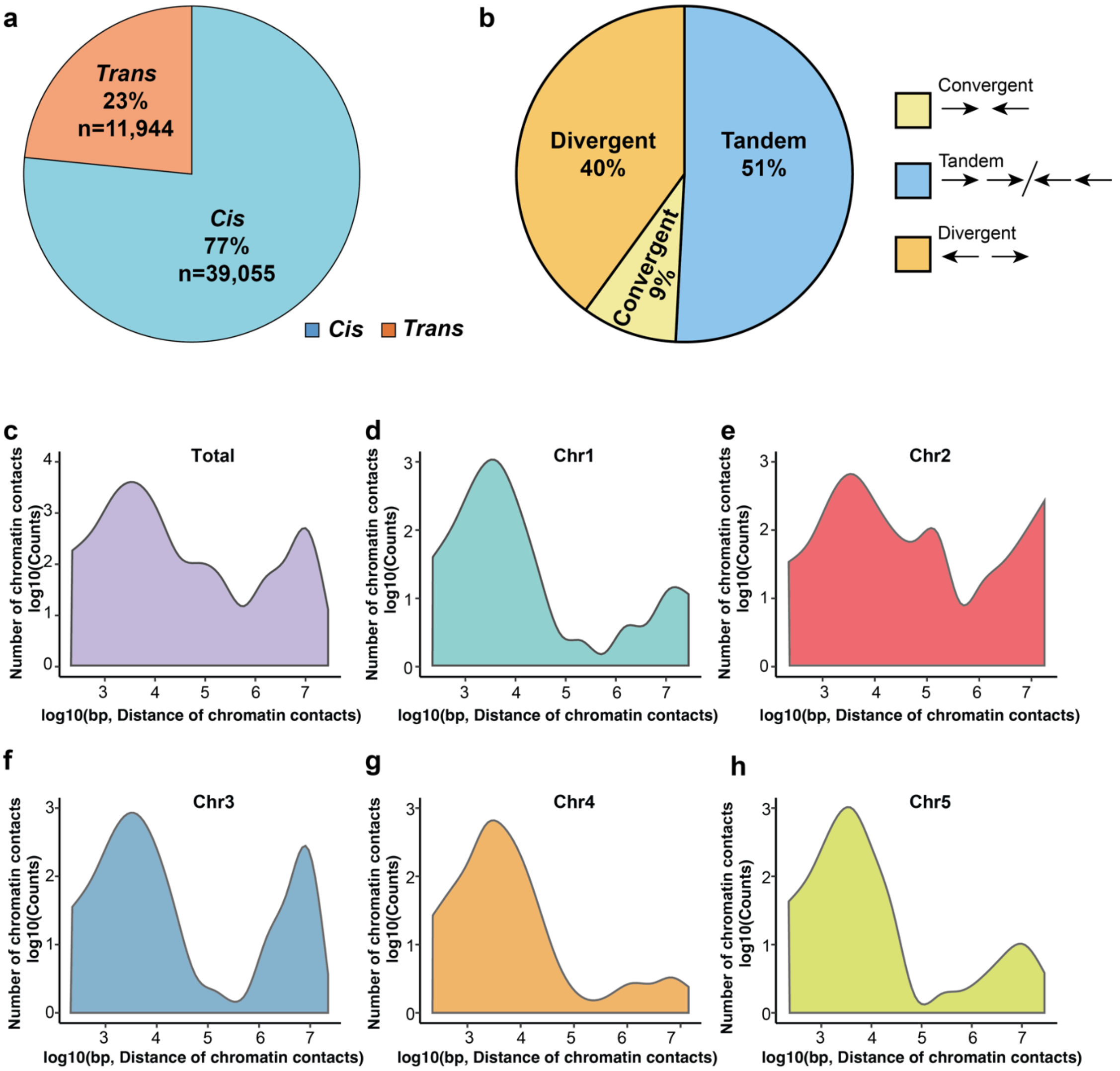

Supplementary Fig. 9 | Overview of PPIs captured in CAP-C libraries.

(**a**) Proportion of *cis* and *trans* PPIs.

(**b**) Proportion of different types of *cis* promoter-promoter interactions.

(**c-h**) The distribution of the distances for all *cis* PPIs in all the chromosomes was plotted respectively: Total PPIs for all five chromosomes (**c**) and PPIs in the individual chromosome (**d-h**).

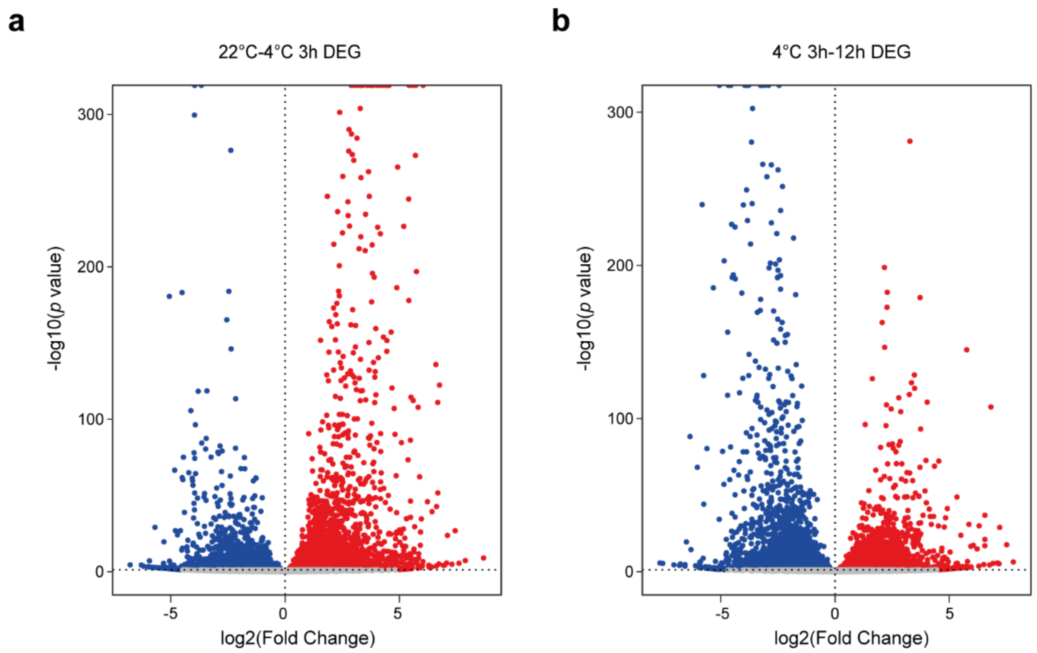

Supplementary Fig. 10 | Differentially expressed genes in response to cold treatments.

(a and **b)** Volcano plots showing differentially expressed genes under 3h cold treatment (4°C) compared with the control at 22°C **(a)** 12h cold treatment compared with the 3h cold treatment **(b)**. All points of interest that display high statistical significance (p value < 0.05) are coloured such that those points representing down-regulated genes are shown in blue, while those representing up-regulated genes are shown in red. In **(a)** the number of the down- and up-regulated genes is 3359 and 3516, respectively. In **(b)** the number of the down- and up-regulated genes is 3386 and 3431, respectively. We conducted differential gene expression analysis using DESeq2, with the default "Wald test" as the testing method.

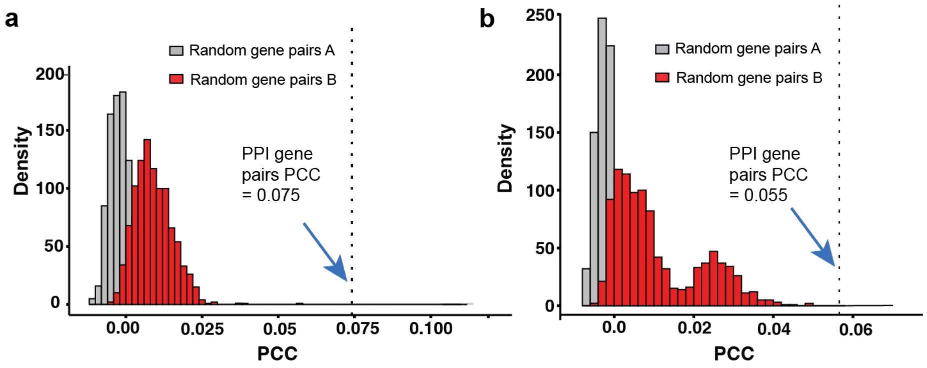

Supplementary Fig.11 | Pearson correlation coefficients of expressions from random gene pairs and PPI connected gene pairs under cold treatment for 3 hours(a) and 12 hours(b). The Pearson correlation coefficients for gene pairs connected by promoter-promoter interaction (PPI) are significantly higher compared to those for randomly selected genes pairs. Randomly selected gene pairs with the same number and similar distance distribution of the PPI connected gene pairs were built as control groups. Random gene pairs A: random gene pairs with the same number; random gene pairs B: random gene pairs with similar distance distribution of PPI gene pairs. Both random procedures were repeated 1000 times. $p < 0.0001$ from t-test.

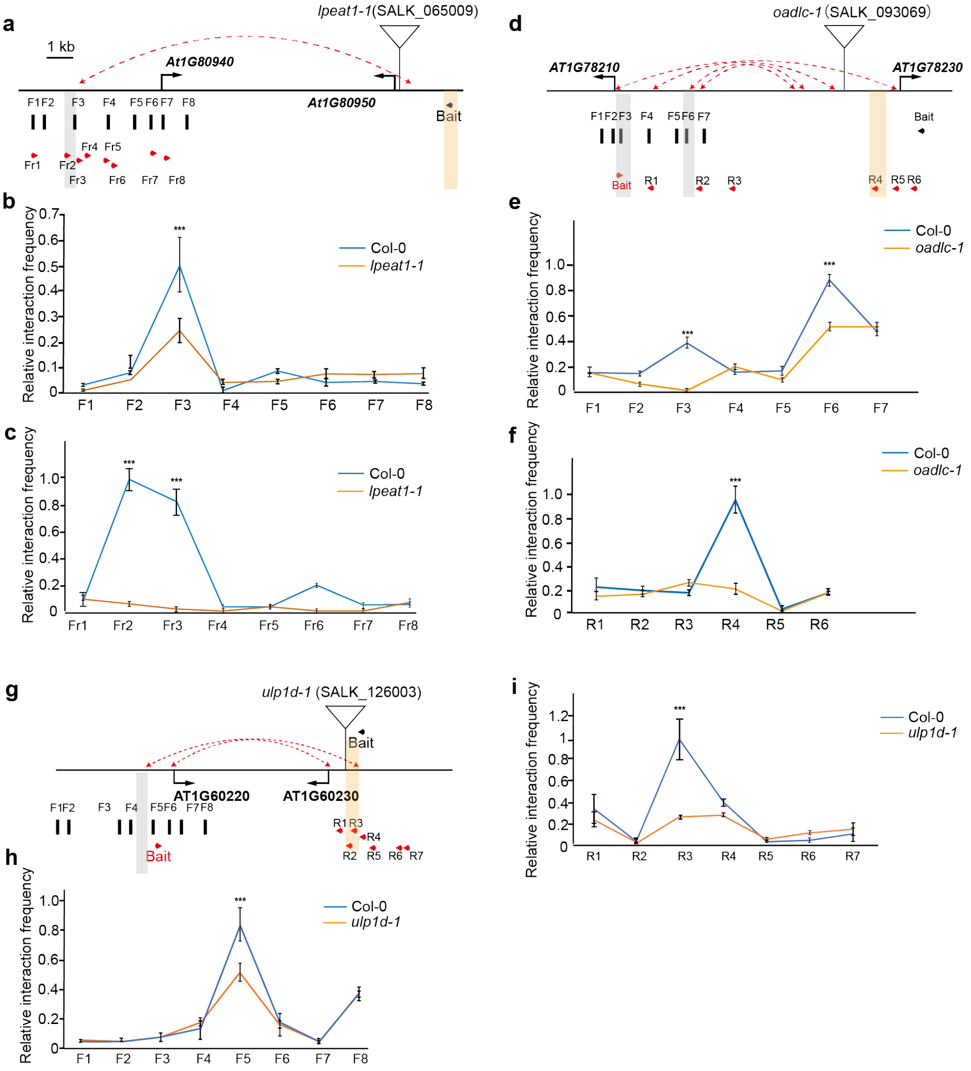

Supplementary Fig. 12 | 3C experiment detection for PPIs in Col-0 and T-DNA insertion mutants. **(a, d and g)** Schematic diagram of gene pairs of *AT1G80940* and *AT1G80950*, the red arc shows the location of the PPI interaction, The triangle indicates the location of T-DNA insertions within the promoter region: *lpeat1-1* (SALK_065009) for *AT1G80950* gene (*LPEAT1*); *oadlc1-1* (SALK_093069) for *AT1G78230* gene (*Outer arm dynein light chain 1*) ; *rssp1-1* (SALK_093069) for *AT1G60230* gene (*Radical SAM superfamily Protein*). The black arrow shows the location of the anchor primer, while black boxes show the locations of the forward primers used in **b, e** and **h**, the red arrow shows the primers used in **c, f** and **i**.

(b, **e** and **h)** 3C experiments show the relative interaction frequency of each fragment with the anchor region, the blue line showing wild-type and the orange line showing the T-DNA insertion mutant.

(c, **f** and **i)** The reciprocal 3C experiment showing the interaction frequency of the confirmed region from b with the reverse primers (shown in red arrows), the blue line showing wild-type and the orange line showing the T-DNA insertion mutant. Three independent biological replicates were assessed, errors

bars indicate SEM; asterisks indicate statistically significant differences using *t*-test; *** indicates $p < 0.001$.

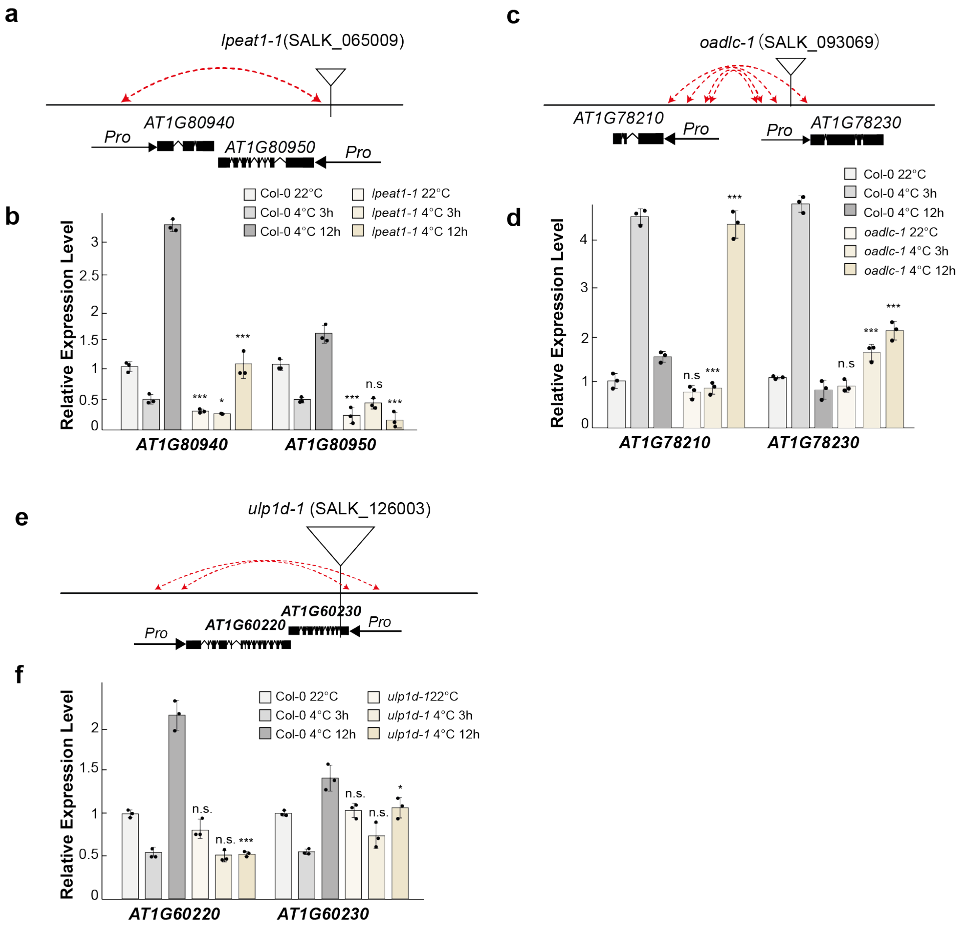

Supplementary Fig.13 | Gene expression detection of genes pairs connected by PPIs in response to cold in wild-type and T-DNA insertion mutants.

(**a**) Schematic diagram of gene pairs of *AT1G80940* and *AT1G80950*, the red arc shows the location of the PPI interaction, The triangle indicates the location of T-DNA insertion.

(b) qRT-PCR analysis of *AT1G80940* and *AT1G80950* in three conditions.

(c) Schematic diagram of gene pairs of *AT1G78210* and *AT1G78230*, the red arc shows the location of the PPI interaction, The triangle indicates the location of T-DNA.

(d) qRT-PCR analysis for gene *AT1G78210* and *AT1G78230* in three conditions.

(e) Schematic diagram of gene pairs of *AT1G60220* and *AT1G60230*, the red arc shows the location of the PPI interaction, The triangle indicates the location of T-DNA insertion within the promoter.

(f) qRT-PCR analysis for gene *AT1G60220* and *AT1G60230* in three conditions. Three independent biological replicates were assessed, errors bars indicate SEM; asterisks indicate statistically significant differences using *t*-test; n.s. not statistically significant; * indicates $p < 0.05$, *** indicates $p < 0.001$.

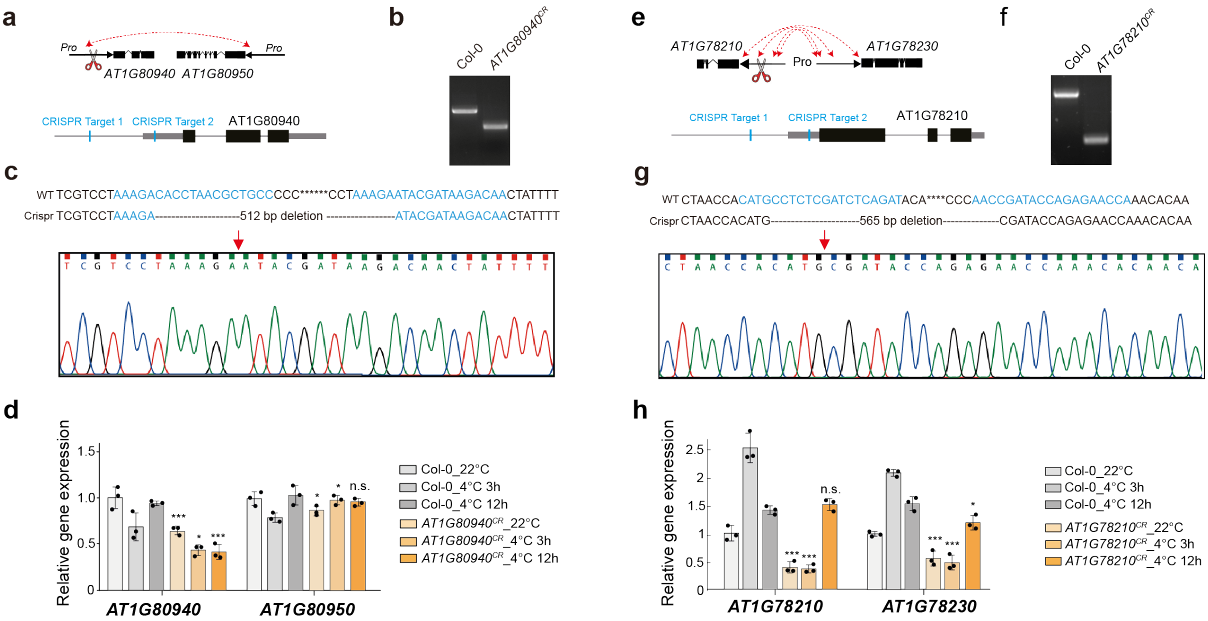

Supplementary Fig. 14 | Gene expression detection of genes pairs connected by PPIs in response to cold in wild-type and CRISPR–Cas9-based deletion mutants.

(**a** and **e**) Schematic diagram of CRISPR-Cas9-based deletions for *AT1G80940* and *AT1G78210* respectively.

(**b** and **f**) PCR analysis confirms the deletion.

(**c** and **g**) DNA sequencing results of the edited band.

(**d** and **h**) qRT-PCR analysis shows gene expression level changes under three conditions. The control Col-0 wild-type and CRISPR-Cas9-based deletion mutants were treated at 22°C and 4°C for 3 and 12 hours, respectively. Three independent biological replicates were assessed, error bars indicate SEM, asterisks indicate statistically significant differences using *t*-test; n.s. not statistically significant; * indicates $p < 0.05$, *** indicates $p < 0.001$.

Reference

1. Wolff J, *et al.* Galaxy HiCExplorer 3: a web server for reproducible Hi-C, capture Hi-C and single-cell Hi-C data analysis, quality control and visualization. *Nucleic Acids Res* **48**, W177-W184 (2020).

2. Potok ME, *et al.* Arabidopsis SWR1-associated protein methyl-CpG-binding domain 9 is required for histone H2A.Z deposition. *Nature Communications* **10**, 3352 (2019).

3. Kindgren P, Ivanov M, Marquardt S. Native elongation transcript sequencing reveals temperature dependent dynamics of nascent RNAPII transcription in Arabidopsis. *Nucleic Acids Res* **48**, 2332-2347 (2020).
