# Supplementary material for: A fine-scale Arabidopsis chromatin landscape reveals chromatin conformation-associated transcriptional dynamics
Source: Nat Commun. 2024 Apr 16;15:3253. doi: 10.1038/s41467-024-47678-7 (PMC11021422; doi:10.1038/s41467-024-47678-7)
Supplement: Supplementary file 3 — Description of Additional Supplementary Files [file 41467_2024_47678_MOESM3_ESM.pdf]

Description of Additional Supplementary Files:

Supplementary Data 1: Mapping statistics of CAP-C libraries.

Supplementary Data 2: Public data used in this study.

Supplementary Data 3: Correlation coefficients between CAP-C chromatin contacts and the enrichment of histone modification.

Supplementary Data 4: Transcription factors in families of bHLH, NAC, and WRKY used in this study.

Supplementary Data 5: Differential expression genes under 3-hour and 12-hour cold treatments.

Supplementary Data 6: Gene ontology analysis of differentially expressed gene pairs containing primed PPIs.

Supplementary Data 7: Stable chromatin contact distance characterizations.

Supplementary Data 8: Enhancer Promoter (E-P) chromatin contact characterizations.

Supplementary Data 9: Enhancer Promoter (E-P) chromatin contact information

Supplementary Data 10: Promoter Promoter Interaction (PPI) characterizations.

Supplementary Data 11: The Gene List of PPIs.

Supplementary Data 12: Primers and plasmids used in this study.
